# The lockdown and its consequences—Perspectives and needs of people at increased risk of severe illness from COVID-19: Results from a qualitative longitudinal study

**DOI:** 10.1007/s00508-021-01979-9

**Published:** 2021-11-24

**Authors:** Erika Mosor, Valentin Ritschl, Margaret R. Andrews, Maisa Omara, Paul Studenic, Gertraud Schaffer, Ernst Leitgeb, Claudia Oppenauer, Linda C. Li, Tanja Stamm

**Affiliations:** 1 Section for Outcomes Research, Center for Medical Statistics, Informatics, and Intelligent Systems, Medical University of Vienna, Spitalgasse 23, 1090 Vienna, Austria; 2 Division of Rheumatology, Department of Internal Medicine III, Medical University of Vienna, Währinger Gürtel 18–20, 1090 Vienna, Austria; 3 Österreichische Rheumaliga (ÖRL), Dorfstraße 4, 5761 Maria Alm, Austria; 4 Austrian Association for Patient Advocacy and Support (AAPAS), Lamprechtgasse 5/7, 1040 Vienna, Austria; 5 Department of Physical Therapy, University of British Columbia, Vancouver, British Columbia Canada; 6 Arthritis Research Canada, Richmond, British Columbia Canada

**Keywords:** Coronavirus, Aged, Chronic disease, Qualitative research, Patient preference

## Abstract

**Background:**

There is a lack of knowledge on how people at increased risk of severe illness from Coronavirus disease 2019 (COVID-19) experienced the infection control measures. This study aimed to explore their perspectives and needs during the coronavirus outbreak.

**Methods:**

A qualitative longitudinal interview study was conducted in Austria during lockdown due to COVID-19 containment and afterwards. People older than 65 years of age and/or affected by a chronic medical condition participated in individual telephone interviews at two time points. Thematic analysis was used to analyze the data and saturation was defined as no new emerging concepts in at least 10 subsequent interviews.

**Results:**

Thematic saturation was reached when 33 individuals (75.8% female, mean age ± standard deviation [SD] 73.7±10.9 years) were included. A total of 44 lower level concepts were extracted and summarized into 6 higher level concepts. They included (i) a general positive attitude toward COVID-19 measures, (ii) challenges of being isolated from the community, (iii) deterioration of health status, (iv) difficulties with measures due to their health condition, (v) lack of physical contact and (vi) lack of information versus overload. Participants suggested environmental adaptations for strengthening resilience in people at increased risk of severe illness from COVID-19.

**Conclusion:**

Strategies and interventions are needed to support people at risk under pandemic conditions. Their perceptions and needs should be addressed to reduce the potential deterioration of health conditions and ensure well-being even during prolonged periods of crisis.

**Supplementary Information:**

The online version of this article (10.1007/s00508-021-01979-9) contains supplementary material, which is available to authorized users.

## Introduction

The novel Coronavirus disease 2019 (COVID-19) spread rapidly worldwide, and the number of cases increased at an accelerated pace [1]. While unexpected changes in daily life affect all people, some persons are more likely to become seriously ill from COVID-19 than others. The Centers for Disease Control and Prevention (CDC) define these people like older adults and people of all ages with certain (chronic) health conditions and severe illness from COVID-19 as an increased risk for hospitalization, intensive care, need for a ventilator and/or death [2]. While the governments of many countries implemented measures to mitigate the pandemic [3], there is a lack of knowledge of how people with an increased risk of severe illness from COVID-19 specifically experienced these measures and under which conditions they were able to best adhere to them.

Recent work identified increased anxiety, depression, insomnia, and stress during the COVID-19 outbreak in all societal groups [4]; however, people who are more likely to be seriously ill from COVID-19 could be even more affected by collateral health damage and negative psychosocial consequences [5]. People from vulnerable groups might be more challenged in daily life and face more severe quarantine consequences, including deteriorated health and well-being, than people from other population groups [6]. Physical distancing measures, such as strict controls of any outdoor activities in response to the COVID-19 pandemic, aim to cut transmission by reducing close social contacts and have been recommended globally to control the community spread of the virus [7]. In Austria, people living in geriatric facilities were particularly protected by the government. Moreover, providers and managers of long-term inpatient care facilities had taken drastic measures, such as instituting visiting bans, curfews, and isolation of residents beyond governmental regulations.

Understanding people’s living environments and preferences at risk of severe illness from COVID-19 in greater depth and incorporating their perspectives into the measures to fight the current or future pandemics are essential aspects of effective, holistic crisis management. Including these individuals’ perspectives and health needs in planning measures could make society more resilient and crisis resistant as a whole in the long term [8]. To the best of our knowledge, few studies have investigated these people’s insights in sufficient depth during a lockdown and the first weeks afterwards.

Therefore, this study aimed to explore the perspectives and needs of people who are more likely to become seriously ill from COVID-19 during different phases of the coronavirus outbreak.

## Subjects and methods

### Study design and participants

A qualitative longitudinal interview study was conducted with individuals from different parts of Austria. Qualitative research is used to explore patients’ perspectives and understand reasons and motivations for behavior as well as preferences and values without imposing a preunderstanding [9]. After the first confirmed cases of COVID-19 in Austria on 25 February 2020, the Austrian government ordered nationwide restriction measures from 16 March 2020 onwards. Until mid-April, public life in Austria remained severely restricted by these measures before the first easing measures were implemented [10].

People older than 65 years and those affected by a chronic health condition, such as heart disease, diabetes, cancer, chronic lung disease and/or any immunodeficiency, were eligible for the current study. Exclusion criteria were insufficient language skills to participate in the interviews (either German or English language) and severe hearing and/or cognitive impairments that would make telephone interviews unfeasible. A maximum variation strategy regarding gender, age, educational level, comorbidity, living situation, and other socio-demographic characteristics was applied [11]. Qualitative research typically uses small sample sizes with a diverse range of participants to explore people’s personal experiences and views on a specific topic in depth [12–14]. Purposive sampling was used as it focuses on particular characteristics of a population of interest and therefore allows identification and selection of information-rich participants [15, 16].

Eligible individuals were identified by patient organizations, geriatric institutions, health professionals and those who participated in the interviews themselves. Appointments were made for remote interviews with participants. The most widely used principle for determining the appropriate sample size and evaluating its sufficiency in a qualitative study is data saturation [17], defined as “the point in data collection when new data no longer bring additional insights to the research questions” [18]. Data analysis started when the first transcripts were available and proceeded parallel to data collection to determine the point of saturation [19]. Despite different approaches in defining data saturation, researchers agreed on some general principles and concepts: no new data, no new themes, no new coding and the ability to replicate the study [20]. In our study, recruitment continued until data saturation was reached, which was defined as no new concepts coming up in at least 10 subsequent interviews [13, 20, 21].

The study was approved by the Ethics Committee of the Medical University of Vienna (EK Number 1388/2020). Participants were informed about the study’s purpose and procedures and provided written informed consent by postal service or email in accordance with the Declaration of Helsinki [22].

### Data collection

Two remote, semi-structured interviews were held with every participant. The research team, including patient research partners (GS, EL), co-developed, piloted and finalized the semi-structured interview guide for the remote interviews [23]. The interview questions focused on people’s perspectives, needs and preferences regarding the impact of COVID-19 measures on their mental and physical health, autonomy, social connectedness, activities, and work/productivity in daily life (Table 1).

**Table 1:** Interview guide

*Introductory—open-ended question*
Since 16 March the Austrian government has implemented numerous measures to mitigate the spread of coronavirus. How have you experienced the time since the implementation of these infection control measures in Austria?
*Semi-structured key questions*
1. What are the effects of coronavirus-related measures on your life/health? (positive/negative)
2. Can you continue to do/decide things that are very important to you?
3. How do you protect yourself and your family/friends?
4. How are you currently in contact with others?
5. What makes you feel better?
6. Would you have needed (more) support during this time?
7. Where/how do you get important information?
*Final questions*
We now come to the end of the interview. Is there anything else you would like to mention about what has been said? Is there anything else that is important that I haven’t asked?

The interviews started during the first lockdown, while the follow-up interviews were conducted in early May 2020 after the government eased measures for the first time. Therefore, the follow-up interview questions were adjusted for the different situation and the initial findings from the first interviews. The first author (EM) experienced in qualitative research data acquisition and analysis, performed the interviews. All interviews were conducted in German, audio recorded, transcribed verbatim, and analyzed centrally in Vienna, Austria, by EM with input from the research team.

### Data analysis

Thematic analysis of qualitative data followed a modified form of “meaning condensation” [23], facilitated by using ATLAS.ti software developed by ATLAS.ti Scientific Software Development GmbH, Berlin, Gemany [24] to organize the data. The analysis comprised the following procedures: in a first step, all transcripts were checked against the audio recordings for accuracy and read several times to gain a broad understanding. After familiarization, data were divided into meaning units (defined as specific parts of the text, a few words or a few sentences with a common meaning). Subsequently, initial codes were assigned to these meaning units. Associated codes were then grouped into lower level concepts. In a final step, the lower level concepts were summarized into higher level concepts. Based on the biopsychosocial model proposed in the International Classification of Functioning, Disability and Health (ICF) and used as a frame of reference in this study [25], the concepts that emerged from the qualitative analysis were used to derive proposals for strengthening resilience that might lead to good individual and community health outcomes [26].

Descriptive statistics were calculated to summarize the characteristics of participants by using R (www.r-project.org). Metric variables were tested for normal distribution. In the case of non-normally distributed data, we depicted medians and interquartile ranges (IQR) in addition to mean values and standard deviations (SD).

### Rigour and accuracy of the study

Several strategies were used to enhance the trustworthiness of the qualitative data [27]. Data triangulation was achieved by interviewing persons of different ages, various diseases and disabilities, rural and urban areas and comparing findings to scientific literature and policy documents throughout the project duration [28]. Reflective memos and debriefing notes were recorded after each interview. Moreover, the first four interviews were independently coded by another experienced investigator (VR). After analyzing all interviews, the results were reviewed and discussed with all researchers and patient research partners (GS, EL), who were not involved in the analysis of the transcripts, until consensus was achieved. Original quotes used for publication were translated from German into English by a bilingual native speaker (MA). Following the translation, data were checked to avoid any translation mistakes by the first author (EM). Finally, we reported the results according to the consolidated criteria for reporting qualitative research (COREQ) checklist ([29]; supplemental table A).

## Results

### Descriptive characteristics of the participants and the data

Thematic saturation (supplemental table B) was reached after including 33 individuals with a mean age ± standard deviation (SD) of 73.7 (±10.9) years. Two telephone interviews with each participant were conducted between 8 April and 15 May 2020. The typical participant was female (75%), had a cardiovascular (39%) and/or musculoskeletal (45%) disease, and was retired (88%), see Table 2. Key characteristics of each participant are depicted in supplemental table C. In total, 27 h and 30 min of interview time were collected (mean duration was 25 min), resulting in 220 pages of transcript. We extracted 44 lower level concepts and summarized them into six higher level concepts (Table 3).

**Table 2:** Characteristics of participants

Participants	Total
***N*** **(%)**	33 (100)
**Women** *n* (%)	25 (75.8)
**Ø Age** (years ±SD) participants	73. (±10.9)
Median (range of age in years; IQR)	76 (46–92;12)
**Personal living situation *n*** **(%)**	
Living alone	7 (21.2)
Living with others	18 (54.5)
Living in a care facility	8 (24.2)
**Diagnosed with the following health condition(s) ***n* (%)	
Diseases of the cardiovascular system	13 (39.4)
Diseases of the digestive system	1 (3.0)
Endocrine, nutritional, and metabolic diseases	6 (18.2)
Diseases of the eyes, ears and related structures	4 (12.1)
Diseases of the musculoskeletal system and connective tissue	15 (45.5)
Diseases of the nervous system	6 (18.2)
Diseases of the respiratory system	7 (21.2)
Diseases of the urogenital system	1 (3.0)
Malignancies	6 (18.2)
None	2 (6.1)
**Employment status ***n* (%)	
Full-time (38.5 h or more per week)	1 (3.0)
Part-time (less than 38.5 h per week)	2 (6.1)
Unemployed	1 (3.0)
Retired	29 (87.9)

*N (%)* total number of participants, *n (%)* number of participants, *Ø Age (±SD)* mean age (standard deviation), *IQR* interquartile range

**Table 3:** Overview of 6 higher level and 44 lower level concepts including original quotes from the study participants

Higher level concepts	Lower level concepts	Example quotes (including sex and age)
6	44	
**1. A general positive attitude**	Being a person who can be well alone	*I don’t dare to say it out loud, but this crisis has now put me at peace. I can have breakfast with my family again. It’s nice to sit at the breakfast table and discuss all kinds of things, and then everyone does something on their own again, but we’re actually happy. *(No 26, female, 66) *I really liked the way the government acted at the beginning of the crisis. It was clear, and one felt that they were saying the same thing and wanted to achieve the same thing. This was great! *(No 4, female, 62) *Very calm and relaxed … we have a lot to read, we have a lot to tell, we have the garden, and sometimes we do nothing at all and just watch the birds or whatever. Very comfortable. *(No 33, female, 71) *I do my exercises every day. What I can do myself, I do. I have a ball, a brush and a Thera-Band at home. *(No 5, female, 64) *We experienced an excellent neighbourhood. To this day, they do the shopping for us and do smaller things, and we get homemade bread, very touching how we are taken care of. *(No 16, female, 78) *You’re alone all the time, and suddenly there’s a knocking, and they [caregivers] are standing out there asking if you want a coffee … and that’s soothing. Very soothing. *(No 9, female, 73, living in a geriatric facility)
	Being able to relax at last	
	Being offered support	
	Coping well with the current situation	
	Experiencing increased connectedness and cohesion	
	Feeling safe and protected	
	Increasing self-responsibility regarding one’s own health	
	Ongoing communication in various ways	
	Opportunity for new activities	
	Supporting others	
**2. Challenges of being isolated from the community**	Being distressed	*At the beginning of the corona crisis, I ordered a toilet seat, and it was so difficult with the company. Finally, they sent the wrong one, and I haven’t been able to exchange this toilet seat yet because I’m afraid to go there and get infected. I haven’t been able to reach anybody either. The phone did not work. I called them a dozen times, and no one answered. *(No 16, female, 78) *What I was afraid of was that I might infect my husband. That was and still is a great fear, I must say. *(No, 31, female, 70) *My children and my husband have been very strict about following government guidelines or measures to not endanger me in any way as a risk patient. And that just creates a certain amount of tension. *(No 23, female, 46) *My husband worked from home; I worked from home, the big ones did their studies from home. The smaller one had to do her homework and in between, the 6‑year-old, who still goes to kindergarten, had to be kept busy. With us, every day was really timed and there was never any ease. *(No 23, female, 46)
	Changes in the living environment	
	Criticism of the behavior of others	
	Dealing with risk of infection	
	Desire for easing COVID-19 measures	
	Increased conflict potential	
	Lack of basic digital literacy skills as an additional obstacle	
	Loss of autonomy	
	Necessary changes in future plans	
	Regular leisure activities no longer possible	
	Worries about a second wave	
**3. Deterioration of health status**	Cancellation of health care services	*Not having therapy was the hardest thing for me during this time. I had to take pills because I couldn’t stand the pain anymore. And I usually take pills very, very rarely. *(No 5, female, 64) *I have someone to help me, too. She does the things that are difficult for me. Now I’ve asked her to stay away. Her husband has gone to work, and she has been shopping over and over again—I just wanted to avoid any risk of contagion.* (No 5, female, 64)
	Increased health problems due to lack of treatment and therapy	
	Reduced availability of doctors and therapists	
**4. Adaptations to improve the implementation of COVID-19 measures**	Difficulties to follow pandemic measures due to disability	*It’s been 2 or almost 3 weeks now since. I’ve tried to organize masks for myself that I can get along with. And it is not easy when you live alone with a disability and should have no contact at all! I have still found no suitable masks or adaptations. It is so difficult to put the “normal” mask on. It takes me 10 minutes or more. As a healthy person, you can’t even imagine that. I can hardly stand it anymore, physically and mentally. *(No 5, female, 64) *I used to have panic attacks. I can’t stand it when my mouth is covered, I have a childhood trauma, and I notice that panic rises inside me when I have to wear the mask for a longer time. *(No 2, female, 47) *You check-in, you lie somewhere in the hallway waiting your turn, you talk to the doctor alone, and it’s sad. It hurts. Usually, there’s always someone there. And now you have to go through this all alone. There are so many things coming at you. And you’re all alone. It’s not easy. *(No 8, female, 52)
	Facing alienation during hospital care	
	Risk to carers due to long waiting time for test results	
**5. Lack of physical contact**	Being concerned about others	*This is an assisted living facility, but I feel like I’m living alone in the house. No life. Nothing at all. People pull away. You just don’t meet anybody. On Saturday I saw some people, and it was the first time after 6 weeks, so I stopped and watched them, mainly because they were talking and I haven’t heard that for a long time. I just stood there and was happy about the kids playing, laughing and being happy. *(No 9, female, 73) *So if I had gotten sick, who would have taken care of my husband? I honestly don’t know what to do. I don’t want my husband in the hospital either. That’s a difficult thing. That’s why I’m so overprotective. *(No 16, female, 78) *Having stupid thoughts, that’s what makes me so depressed. If I had something to do, if I had my husband—as a couple, you always have something to talk about, and that protects you, and that’s missing … and if I can’t speak to anyone, then the negative thoughts come instead.* (No 9, female, 73)
	Implementation of social/physical distancing measures	
	Importance of being close to each other	
	Increasing loneliness and depression	
	People have been abandoned	
	Restrictions despite palliative care	
	Use of protective measures like masks	
**6. Lack of information versus overload**	Feeling fooled by the government/loss of credibility	*Despite the ban, I still had my son brought to us every Monday by the transport service. It was important for me because the therapies did not take place, my sister did not visit him, and he has no one else. But he needs someone to talk to, someone to understand him. He said I was breaking the law. Later, the government said that this was not against the law at all. That was not right! *(No 28, female, 81) *At the end of the day, when the measures were eased, it was said: “You could have always met the family in private spaces; there would have been no objections.” I didn’t like that, because I simply want to be treated as an adult and not like a fool. Their credibility suffers, and I think that is a pity. *(Nr 4, female, 62) *It is such a strange situation, on the one hand, you are constantly told how to behave, and on the other hand, you realise that it doesn’t work that way in everyday life, so we don’t work that way, our life doesn’t work that way. *(No 23, female, 46)
	Information status and channels used	
	Measures affecting working conditions	
	Need to justify own decisions regarding COVID-19 measures to others	
	Perspective regarding government work	
	Reasons for accepting the measures	
	Relief through the easing of measures	
	Trust in information provided	
	Weighing up the amount of news so as not to be misinformed or overwhelmed, bored or frightened	
	Worry about the economic situation	

### A general positive attitude

The participants expressed a general positive attitude towards measures that have been taken to mitigate the spread of COVID-19. Some people felt particularly well protected under strict home isolation and appreciated positive aspects of the crisis that helped them better cope with this exceptional situation. They had been offered support from their families, carers, neighbours, and other people they had not known before. Several participants even experienced increased connectedness and cohesion in times of the pandemic, like a 73-year-old woman (No 9) who said:

And still, I feel better now, because I have much more contact with people, even if it is only by phone. They ask me how I am, what I’m doing. It’s good for my well-being.

Moreover, some people found more time to relax, tried out new activities during home isolation, and supported others to better deal with this exceptional situation. In the absence of regular treatment and therapy, several study participants (8,24%) reported increased self-management regarding their health.

### Challenges of being isolated from the community

Strict home or community isolation was described as an experience of being “captured and imprisoned”. A 90-year-old man (No 19) living in a geriatric facility experienced this situation as follows:

We all yearn to sit at a table at noon and talk. To be locked up in a room and eating alone is kind of torture.

A woman (No 11) at the age of 79 years who lived independently in an assisted-living facility described a situation in which she felt at the mercy of others as follows:

They lock us up in here and won’t let us out. Security is down there around the clock. We have to stay in our apartments and can’t have any contact with the outside world, except for by phone. That’s no kind of life!

### Deterioration of health status

Almost one third of the participants (10, 30.3%) reported a significant deterioration in their health status caused by reduced routine care and therapy during the lockdown. In their view, health care changed dramatically from one day to the next, and there were only a few telehealth offers. A 64-year-old woman (No 5) with a physical disability said:

I notice that the longer this goes on, the more difficult it becomes, physically and mentally. Especially physically. My body doesn’t work that way anymore. I stumble through the cramps more often, although I really do my exercises umpteen times a day at home and I am really active, but I can’t compensate for this alone, I lack the therapy.

People with already existing health problems lacked medical care, treatment, and supply of assistive devices and regular personal contact with their physicians and therapists during this period; however, some people cancelled therapeutic interventions and home care services themselves out of fear of infection with SARS-CoV‑2, which led to an enormous additional burden in everyday life. One woman (No 28) at the age of 81 years, suffering from multiple health conditions herself, described the reasons for cancelling her husband’s care:

We were afraid; the carers have children or go to people who are even worse off than my husband. […] Now I just have to find a way to wash him.

### Adaptations to improve the implementation of COVID-19 measures

Some people reported that particular adaptations of COVID-19 measures would have made it easier to implement them. More than half of the participants (18, 55%) in this study had problems following basic COVID-19 protection measures due to their functional limitations in daily life. They could not wear masks because of physical impairment, had panic due to past traumatic experiences when wearing masks or experienced breathing difficulties. Moreover, participants described barriers to implementing basic hygiene measures, such as disinfection stands which are often not accessible for people using wheelchairs or persons of small stature. Like a woman (No 5) aged 64 years with restrictions in her mobility, some people also experienced difficulties in enacting physical distancing because of additional support needs.

I need some personal assistance while walking because I feel much safer then, but this is not allowed. So, my only option is the wheelchair or not playing by the rules.

Others also experienced difficulties in communication due to the use of face masks and Plexiglas barriers. One female participant (No 8), who had to stay in hospital for a few days, talked about washbasins that she could not reach:

I am 106 cm tall and have difficulty reaching the sink in the hospital. And then they always tell you to wash your hands. I asked if they could please bring me a bottle of disinfectant, but they said, “No, we can’t do that—no distribution to patients”.

### Lack of physical contact

Although most participants in this study (25, 76%) have stayed connected and maintained their social networks in a non-face-to-face way, they still suffered from the ban on meeting others in person. They emphasized the importance of personal contact and closeness. From their perspective, prohibiting physical contact and closeness led to social isolation and deprivation, which constituted a massive threat to people’s physical and mental health. They reported increased confusion, malnutrition, sadness, increasing desperation and lack of physical activity among relatives and friends they were no longer allowed to visit. They felt that especially older adults, people with cognitive impairment, hearing problems, and those who were seriously ill or dying, have been abandoned during the first weeks of the pandemic, and nobody cared. A woman (No 3), 65 years old, gave the following example:

… and then people like my aunt at 103 years die due to isolation or loneliness, or depression because they are so alone. This just can’t go on! Something must be done quickly.

### Lack of information versus overload

People reported that they repeatedly had to weigh up how much information they needed not to be misinformed or overloaded or become highly concerned about their own health or bored. A 75-year-old man (No 29) expressed his experience in the following statement:

… it gets on my nerves, this constant information about things I already know. I think there are already more experts than corona patients. So I’m switching off immediately.

Some participants felt that the information they received was not sufficiently concise and relevant to their underlying conditions. This was perceived as a potential risk to their personal health. For example, a woman (No 23) at the age of 46 years with severe lung disease, the information provided by the government did not meet her needs.

I think this information about the course of the disease was somehow minimal. I would like to have a more informed discussion of the facts. Instead, we were treated as minors.

Only one third of the participants (11 out of 33) were concerned about possible economic consequences that might affect them personally.

### Longitudinal perspective

Almost all participants welcomed the easing of measures that took place between the first and subsequent telephone interviews. Over time, however, they experienced (i) an increasing concern about misconduct by others regarding compliance with infection control measures, (ii) increased levels of loneliness and depression, (iii) a feeling of being overwhelmed or bored by the amount of information provided about the pandemic and the measures taken to mitigate its effects and (iv) difficulties in adhering to specific measures due to their functional limitations/disabilities when they started to leave home again.

### ICF mapping and environmental adaptations from the perspective of the participants

The higher level concepts were mapped to 29 ICF categories in total; 4 (14%) related to body functions and 1 (3%) to body structure, 17 (59%) to activities and participation, and 7 (24%) to environmental factors (Table 4). The pandemic and the measures to mitigate its impact thus affected all components of functioning and health specified in the ICF classification.

**Table 4:** Higher level concepts derived from the qualitative analysis, mapped ICF categories [33], and corresponding environmental adaptations and personal support needs

Higher level concepts	Mapped ICF categories	Environmental adaptations and personal support needs from the perspective of the participants
**A general positive attitude**	d230 Carrying out daily routine d240 Handling stress and other psychological demands d2402 Handling crisis d350 Conversation d360 Using communication devices and techniques e310 Immediate family e320 Friends e325 Acquaintances, peers, colleagues, neighbours, and community members e355 Health professionals	*Promotion of healthy behavior and education in self-management and the use of digital technology for communication* Educate people on how best to stay physically active; connect with others by using new technologies such as telephone consultations, text messaging and video conferencing; and manage the stress of daily life through the challenging times of a crisis
**Challenges of being isolated from the community**	d460 Moving around in different locations d470 Using transportation d845 Acquiring, keeping and terminating a job d910 Community life d920 Recreation and leisure d930 Religion and spirituality d940 Human rights	*Information to avoid feelings of helplessness and outside control* Provide detailed information for people affected by home or community isolation about the necessity of social/physical distancing policies to avoid feelings of helplessness and loss of control. If necessary, the information would have to be given in a personal conversation in an easily understandable way
**Deterioration of health status**	b280 Pain b710 Mobility of joint functions b735 Muscle tone functions b770 Gait pattern functions s 750 Structure of lower extremity e580 Health services, systems and policies	*Delivery of routine health service at any time* Undertake immediate action to improve care and support to prevent any deterioration in health status resulting from isolation measures for people at risk (such as chronically ill, disabled, old, frail, or cognitively impaired). Therefore, routine health service delivery may need to be adapted to be reliably continued for people at higher risk of serious illness from COVID-19, even during a future crisis
**Adaptations to improve the implementation of COVID-19 measures**	e1101 Drugs e1151 Assistive products and technology for personal use in daily living	*Identification and elimination of obstacles and barriers to best follow the infection control measures* Implement points of contact to best support people and address their specific needs to maintain their health, safety, and independence in the community throughout the COVID-19 outbreak and related health emergencies
**Lack of physical contact**	d710 Basic interpersonal interactions d720 Complex interpersonal interactions	*Assurance of personal contact if needed* Raise awareness that especially older people and people with underlying medical conditions should never be left alone for long periods. This might have negative consequences for their health and well-being. The involvement of family members and other support networks should be allowed even in times of crisis with care and consideration; without preventing an encounter per se
**Lack of information versus overload**	d310 Communicating with—receiving—spoken messages d320 Communicating with—receiving—formal sign language messages d325 Communicating with—receiving—written messages	*Incorporation of people’s perspective in decision-making* Involve representatives of people at increased risk of severe illness from COVID-19 in expert groups to identify context-relevant essential information during an outbreak

This highlights the importance of adaptations in the physical, social and attitudinal environment in which people live and conduct their lives for better functioning and health of individuals. The participants mentioned several actions that could help increase compliance with the measures (Table 4, far right column). In their opinion, some environmental adaptations, such as more accessible holders for disinfectants in offices and shops for wheelchair users, and user-friendly digital technology to overcome social isolation and maintain access to health care providers despite lockdown, could have a significant impact on the extent to which people at increased risk of severe illness from COVID-19 comply with risk reduction measures. Those actions could help to prevent collateral health deterioration in this group of people.

## Discussion

The present study comprehensively explored the experiences and needs of people at increased risk of severe illness from COVID-19 in response to the early phases of the pandemic. The actions that were implemented during the lockdown had significant positive and negative impacts on the functioning and health of the selected population. Although the initial infection control measures taken by the government were generally accepted and valued by older adults and people with underlying chronic health conditions, individuals also expressed challenges in daily life and potential negative consequences of the lockdown that may have been given too little consideration. These collateral damages to the population at risk have not been well understood thus far. Studies have mainly focused on health care providers [30–32], often not considering the needs of people at increased risk of severe illness from COVID-19.

Some participants in our study reported additional limitations in their functioning in daily life caused by the infection control measures [33]. They experienced reduced access to health care services, such as hospitals and outpatient clinics, and the suspension of many health care interventions. Moreover, some of them would have needed specific exemptions to follow the infection control measures properly. Especially in high strain on the health care system, it is crucial to ensure that all population groups receive good care. Physical and mental health are essential for resilience throughout the COVID-19 outbreak and related health emergencies [34]. Health in all policies requires equity and includes strategies to achieve the best health outcomes and a sustainable health care system for everyone in our society [35]. Participants in this study were convinced that incorporating their perspectives into the design of health care in the context of the pandemic would make an important contribution to societal health ensuring adequate compliance with the specific measures taken.

People also pointed out that constant social contact with people with cognitive, mental, psychosocial and/or physical disabilities or chronic health conditions is urgently needed to prevent a deterioration in health and promote well-being despite times of crisis. In their work, Cacioppo et al. pointed out that social isolation impairs older people’s mental and cognitive health and is also accompanied by a significantly higher risk of mortality [36], underpinned by a meta-analytic review done by Holt-Lunstad et al. [37]. Using existing support networks with care and considering the risk of infection might help overcome the disadvantage of this vulnerable group even in times of a pandemic.

Interestingly, and in contrast to population-based surveys on the population’s health status during pandemics in other countries [38], participants in this study were less concerned about possible financial uncertainties caused by the crisis; however, most of them were retired and trusted the existing social and health system, which might be different for people living in countries with a less established or even not existing safety net.

In their work, Teti et al. concluded that the needs and preferences of people at risk must be studied in depth to plan health care efficiently [8]. With this study we provided comprehensive longitudinal data but remotely collected, which might be regarded as a limitation of our work. Nevertheless, our findings highlight the need for further research to understand the role of resilience in managing the extraordinary challenges due to COVID-19 among people at risk of severe illness and to explore interesting changes in the experience of the applied measures over time in more depth. The evidence provided will support the formulation of preparedness and response strategies targeted and based on the preferences of this vulnerable group of individuals. Targeted strategies and interventions such as maintaining access to health care providers, facilitating the use of digital technology to overcome social isolation, and adapting the environment are needed to increase compliance with risk reduction measures, prevent collateral health deterioration in this group, and ensure well-being even during prolonged periods of crisis.

## Conclusion

Participants in this study have identified several opportunities to promote and protect the health and well-being of people at higher risk of serious illness from COVID-19 and reduce health inequity. The study results might guide policy and health care providers to minimize direct and indirect harm and effectively support people living under demanding conditions in such a pandemic. In addition, the knowledge gained could be used to revise outbreak response activities and develop action plans and interventions for future waves of infection or pandemics to promote best and protect population health and well-being in different population groups.

## Supplementary Information

Supplemental tables A, B and C
