# Supplementary material for: The lockdown and its consequences—Perspectives and needs of people at increased risk of severe illness from COVID-19: Results from a qualitative longitudinal study
Source: Wien Klin Wochenschr. 2021 Nov 24;133(23-24):1255–64. doi: 10.1007/s00508-021-01979-9 (PMC8612388; doi:10.1007/s00508-021-01979-9)
Supplement: Supplementary file 1 — Supplemental tables A, B and C [file 508_2021_1979_MOESM1_ESM.docx]

**APPENDIX**

**Supplemental table A.** COREQ (COnsolidated criteria for REporting Qualitative research) Checklist

| **Topic** | **Item no.** | **Guide Questions/’Description** | **Reported in section** |
| --- | --- | --- | --- |
| **Domain 1: Research team and reflexivity** | | | |
| **Personal characteristics** | | | |
| Interviewer/facilitator | 1 | Which author/s conducted the interview or focus group? | The first author (…), female, PhD, background in occupational health and health science, with experience in qualitative research data acquisition and analysis, performed the interviews. |
| Credentials | 2 | What were the researcher's credentials? E.g. PhD; MD |  |
| Occupation | 3 | What was their occupation at the time of the study? |  |
| Gender | 4 | Was the researcher male or female? |  |
| Experience and training | 5 | What experience or training did the researcher have? | The interviewers had years of experience in the field of qualitative research. This is reported in the methods section. |
| **Relationship with participants** | | | |
| Relationship established | 6 | Was a relationship established prior to study commencement? | The study participants were approached if they wanted to by …. |
| Participant knowledge of the interviewer | 7 | What did the participants know about the researcher? e.g. personal goals; reasons for doing the research | The researchers introduced themselves and informed the participants comprehensively about the goals and the content of the study. All participants gave oral and written consent to be included in this study. This is reported in the methods section, as well as in the declaration section.  Reasons for exploring personal experiences during the COVID-19 pandemic is reported in the background section. |
| Interviewer characteristics | 8 | What characteristics were reported about the interviewer/facilitator? e.g. Bias; assumptions; reasons and interests in the research topic |  |
| **Domain 2: Study design** | | | |
| \| **Theoretical framework** \| \| --- \| | | | |
| Methodological orientation and theory | 9 | What methodological orientation was stated to underpin the study? e.g. grounded theory; discourse analysis; ethnography; phenomenology; content analysis | We undertook a qualitative study using thematic analysis for analysing the qualitative interview data. This is reported in the methods section. |
| **Participant selection** | | | |
| Sampling | 10 | How were participants selected? e.g. purposive; convenience; consecutive; snowball | We included individuals who agreed to participate in this study. The selection criteria and are reported in the methods section. |
| Method of approach | 11 | How were participants approached? e.g. face-to-face; telephone; mail; email | All participants were contacted by phone. Those wishing to participate were asked for a suitable time for conducting the telephone interviews. This is reported in the methods section. |
| Sample size | 12 | How many participants were in the study? | Information about the sample and sample size is provided in the results section and in table 2. All individuals that were willing to participate in the study finished the interviews. |
| Non-participation | 13 | How many people refused to participate or dropped out? Reasons? |  |
| **Setting** | | | |
| Setting of data collection | 14 | Where was the data collected? e.g. home; clinic; workplace | The interviews were conducted as telephone interviews. Only the participant and … were present. |
| Presence of non-participants | 15 | Was anyone else present besides the participants and researchers? |  |
| Description of sample | 16 | What are the important characteristics of the sample? e.g. demographic data; date | The baseline characteristics of the sample are reported in table 2. |
| **Data collection** | | | |
| Interview guide | 17 | Were questions; prompts; guides provided by the authors? Was it pilot tested? | As reported in the methods section, the interview guide was developed and adapted together with the patient research partners. |
| Repeat interviews | 18 | Were repeat interviews carried out? If yes; how many? | Two telephone interviews per person were conducted, one during the first lockdown and the second one after easing the COVID-19 measures. This is reported in the methods section. |
| Audio/visual recording | 19 | Did the research use audio or visual recording to collect the data? | Interviews were all audiotaped and transcribed verbatim, and debriefing notes were taken after each interview. This is reported in the methods section. |
| Field notes | 20 | Were field notes made during and/or after the interview or focus group? |  |
| Duration | 21 | What was the duration of the interviews or focus group? | In total, 27 hours and 30 minutes of interview time were collected (mean duration of 25 minutes). This is reported in the result section. |
| Data saturation | 22 | Was data saturation discussed? | Data saturation was defined as no new concepts coming up in at least ten subsequent interviews and reported in the methods section and the results section. Details are depicted in the last row of supplemental table B. |
| Transcripts returned | 23 | Were transcripts returned to participants for comment and/or correction? | Transcripts were not returned to the participants for comments and/or corrections. However, two patient researcher partners (… and …) reviewed the data analysis to ensure that the results comprehensively cover participants' views. This is reported in the methods section. |
| **Domain 3: analysis and findings** | | | |
| **Data analysis** | | | |
| Number of data coders | 24 | How many data coders coded the data? | The data coding and analysis was primarily done by …; Vienna; Austria, with input from the research team. This is reported in the methods section. |
| Description of the coding tree | 25 | Did authors provide a description of the coding tree? | A detailed description of the analysis steps is provided in the methods section. |
| Derivation of themes | 26 | Were themes identified in advance or derived from the data? | The identified themes were derived from the interview data. This inductive data analysis is described in detail in the methods section. |
| Software | 27 | What software; if applicable; was used to manage the data? | The qualitative data analysis was facilitated by using ATLAS.ti software [24]. |
| Participant checking | 28 | Did participants provide feedback on the findings? | Two patient research partners (… and …) provided feedback on the findings (reported in the methods section). |
| **Reporting** | | | |
| Quotations presented | 29 | Were participant quotations presented to illustrate the themes/findings? Was each quotation identified? e.g. participant number | Quotations are presented in the results section and table 3. Each quotation was at least indexed using participants' sex and age. |
| Data and findings consistent | 30 | Was there consistency between the data presented and the findings? | We endeavoured to ensure consistency between the data presented and the findings by using quotes to support our interpretations/findings. Please see the results section and tables 3 and 4, as well as supplemental table B. |
| Clarity of major themes | 31 | Were major themes clearly presented in the findings? | We described our findings, including original quotes from the participants in the results section. In addition, we presented the qualitative coding scheme (higher-level and lower-level concepts including original quotes from the study participants) in table 3.  Table 4 shows aspects of functioning in times of a pandemic based on the ICF and examples of domains being affected, and targeted actions derived from the analysis of interview data for strengthening resilience in people at increased risk of severe illness from COVID-19.  Supplemental table B shows the diversity of cases and minor themes that also came up during the interviews and the subsequent data analysis. |
| Clarity of minor themes | 32 | Is there a description of diverse cases or discussion of minor themes? |  |

**Supplemental table B.** Lower-level concepts mentioned by the participants during the initial and the follow-up interviews; the last column shows the frequency of lower-level concepts and the percentage; the last row shows that no 'new' lower-level concept came up in the last ten initial interviews; indicating that thematic saturation was reached after including 33 participants.

| **Higher-level concept** | **Lower-level concept** | **Lower-level concepts mentioned by the participants** | | | | | | | | | | | | | | | | | | | | | | | | | | | | | | | | |  |
| --- | --- | --- | --- | --- | --- | --- | --- | --- | --- | --- | --- | --- | --- | --- | --- | --- | --- | --- | --- | --- | --- | --- | --- | --- | --- | --- | --- | --- | --- | --- | --- | --- | --- | --- | --- |
| **6** | **44** | **1** | **2** | **3** | **4** | **5** | **6** | **7** | **8** | **9** | **10** | **11** | **12** | **13** | **14** | **15** | **16** | **17** | **18** | **19** | **20** | **21** | **22** | **23** | **24** | **25** | **26** | **27** | **28** | **29** | **30** | **31** | **32** | **33** | **Total (%)** |
| 1. **A general positive attitude** | being a person who can be well alone | N | + |  |  |  |  |  |  |  | + |  | # |  | + |  |  |  | + |  |  |  |  |  |  |  | + |  |  |  |  |  |  |  | 7 (21.2) |
|  | being able to relax at last |  |  |  |  |  |  |  |  |  |  |  |  |  | N |  |  |  |  |  |  |  |  |  |  |  | + |  |  |  |  | + |  | + | 4 (12.1) |
|  | being offered support | N |  |  |  | + | + | + | + | + | + |  | + |  | + | + | + | + |  | + | + | + | + |  |  |  | + | + | + |  |  | + |  |  | 20 (60.6) |
|  | coping well with the current situation | N | + |  | + | + |  |  | + |  |  |  | # | + | + |  |  |  |  |  | + | + | # |  | + | + | + | + | + |  | + | + | + | + | 20 (60.6) |
|  | experiencing increased connectedness and cohesion |  | N |  |  | + |  |  |  | + |  |  |  | + |  |  |  | + |  |  | + |  |  |  |  |  | + | + |  | + |  | + |  |  | 10 (30.3) |
|  | feeling safe and protected |  |  |  |  |  |  |  |  |  |  |  | **N** | **+** |  |  |  |  |  | + |  |  |  |  |  | + |  |  |  |  | + |  |  |  | 5 (15.2) |
|  | increasing self-responsibility regarding one's own health |  |  |  |  | N |  | + |  |  |  |  |  |  | + |  |  |  |  |  | + |  |  |  | + |  | + |  |  |  |  | + |  | # | 8 (24.2) |
|  | ongoing communication in various ways | N | + | + | + | + | + | + |  | + | + | + | + |  | + | + | + |  |  |  | + | + | + | + | + |  | + | + | + | + |  | + |  | + | 25 (75.8) |
|  | opportunity for new activities | N | + |  |  | + | + | + | # | + | + |  |  | # |  |  |  |  |  |  | # |  | + |  |  |  | + |  |  |  |  |  |  | + | 13 (39.4) |
|  | supporting others |  |  | N |  | + |  | + |  |  |  |  |  |  |  |  |  |  |  |  |  |  |  | # |  |  | + | + | + |  |  |  |  |  | 7 (21.2) |
| 1. **Challenges of home or community isolation** | being distressed |  |  |  |  |  |  |  |  |  |  | **N** |  |  |  |  | + |  | + |  | # |  | + | + |  |  | + |  |  | # |  | + |  | + | 10 (30.3) |
|  | changes in the living environment |  | **N** |  |  |  |  |  | **+** |  |  |  |  |  |  |  |  |  |  |  |  |  |  | + |  |  |  |  | # |  |  |  |  |  | 4 (12.1) |
|  | criticism of the behaviour of others |  |  |  |  |  |  |  |  |  |  |  |  |  |  |  |  |  |  |  |  |  | **N** | # |  |  |  |  |  | + | + | + | + |  | 6 (18.2) |
|  | dealing with risk of infection | **#** | **N** | **+** | **+** | **#** | **+** |  | **+** |  | **+** |  | **+** |  | + | + | + |  | + |  | # | + | + | # |  | + | # |  | + |  |  | # |  | + | 22 (66.7) |
|  | desire for easing COVID-19 measures | **N** |  | **+** |  | **+** |  | **+** | **+** | **+** | **+** | **+** | **#** | **+** |  |  |  |  |  |  |  | + |  | # |  |  |  |  |  | + | # |  |  |  | 14 (42.4) |
|  | increased conflict potential |  | **N** |  |  |  |  |  |  |  |  |  |  |  |  |  |  |  |  |  |  |  |  | + |  |  |  |  |  |  |  |  |  | + | 3 (9.1) |
|  | lack of basic digital literacy skills as additional obstacle |  |  |  |  |  |  |  |  |  |  |  |  |  | **N** | + |  |  | + |  |  |  |  |  |  |  |  |  |  |  |  |  |  |  | 3 (9.1) |
|  | loss of autonomy |  |  |  |  |  |  |  |  | **N** | + | + | **+** | **#** | + |  |  |  | + | + |  |  |  | + |  |  | + | + | + |  | + |  |  |  | 13 (39.4) |
|  | necessary changes in future plans | **N** |  | **+** | **#** | **+** | **#** | **+** |  |  |  |  |  |  |  |  | + | + |  |  |  |  | # |  | + |  | + |  |  |  |  |  |  | + | 12 (36.4) |
|  | regular leisure activities no longer possible | **N** |  |  |  | **+** | **+** | **+** |  |  |  | + |  |  |  | + | + |  |  |  |  | + |  |  | # | + | + |  |  |  | + | + |  | + | 14 (42.4) |
|  | worries about a second wave |  | **#** |  |  |  |  |  |  |  | # |  | **#** | **N** |  | # |  |  | + |  | + | + | + | # |  |  | + |  |  | + | + | + | + | + | 16 (48.5) |
| 1. **Deterioration of health status** | cancellation of health care services |  |  |  |  | **N** |  |  | **+** | **+** |  |  |  |  |  | + |  |  |  |  |  |  |  |  |  |  |  |  | + |  |  |  |  |  | 5 (15.2) |
|  | increased health problems due to lack of treatment and therapy |  |  | **N** |  | **+** |  | **#** | **+** |  |  |  | **+** |  |  |  | + |  | # | # |  | + |  |  | + |  |  |  |  |  |  |  |  |  | 10 (30.3) |
|  | reduced availability of physicians and therapists | **N** | **+** | **+** |  | **+** | **+** |  | **+** | **+** |  |  | **#** |  |  | + | + | + | # |  |  | + | + |  |  |  |  | + | + | + |  |  |  | + | 18 (54.5) |
| 1. **Adaptations of measures to improve implementation** | difficulties to follow pandemic measures due to disability | **N** | **+** | **+** | **+** | **#** |  | **#** | **+** | **#** |  |  | **#** | **+** |  | + | + |  |  |  | + |  | # | + |  |  | + |  |  |  |  | # |  | + | 18 (54.5) |
|  | facing alienation during hospital care |  |  |  |  |  |  |  | **N** |  |  |  |  |  |  |  |  |  |  |  |  |  |  |  |  |  |  |  |  |  |  |  |  |  | 1 (3.0) |
|  | risk to carers due to long waiting time for test results |  |  |  |  |  |  |  | **N** |  |  |  |  |  |  |  |  |  |  |  |  |  |  |  |  |  |  |  |  |  |  |  | + |  | 2 (6.1) |
| 1. **Lack of physical contact** | being concerned about others |  | **N** | **+** | **+** |  |  |  |  |  |  |  |  |  |  |  |  |  |  |  |  |  |  |  |  |  |  |  |  |  |  | + |  | + | 5 (15.2) |
|  | implementation of social/physical distancing measures | **N** | **+** | **+** | **+** |  | **+** |  | **+** | **+** | **+** |  |  |  |  |  |  |  |  |  | + |  |  | + | + |  |  | + | + | + | + | + | + |  | 17 (51.5) |
|  | importance of being close to each other |  | **N** | **+** | **+** | **+** |  | **#** |  | **+** |  |  | **+** | **+** |  |  | + | + |  | + | + | + | + | + |  |  |  |  | + | + | + | + | + | + | 21 (63.6) |
|  | increasing loneliness and depression |  |  |  |  | **#** |  | **#** |  | **N** |  | **#** |  | **#** |  |  | + |  |  | # |  |  | + |  |  |  | # |  | # |  |  |  |  |  | 10 (30.3) |
|  | people have been abandoned |  |  | **N** | **+** | **+** | **+** | **+** |  |  | **+** |  |  |  | + |  |  |  |  |  |  |  |  |  |  |  | + |  | + |  | # |  |  | + | 11 (33.3) |
|  | restrictions despite palliative care |  |  | **N** |  |  |  |  |  |  | **+** |  |  |  | + |  |  |  |  |  | # | # |  |  |  |  |  |  |  |  |  |  |  |  | 5 (15.2) |
|  | use of protective measures like masks | **N** | **+** | **+** | **+** | **+** |  | **#** | **+** |  | **+** | **+** | **+** | **+** | + |  |  |  | + | + | # |  | + | + | + | + | + | + | + |  | + | + |  |  | 24 (72.7) |
| 1. **Lack of information versus overload** | feeling fooled by the government |  |  | **#** | **#** |  |  |  |  |  |  |  |  |  |  |  |  |  |  |  |  |  |  | **N** |  |  |  |  | + |  |  |  | + |  | 5 (15.2) |
|  | information status and channels used | **N** | **+** | **+** |  | **+** | **+** | **+** |  | **+** | **+** |  | **+** |  | + | + | + |  |  | + | + |  | + |  | + | + |  |  |  |  |  | + |  |  | 18 (54.5) |
|  | measures affecting working conditions |  | **N** |  |  |  |  | **+** | **+** |  |  |  |  |  |  |  |  |  |  |  |  |  |  | + |  |  | # |  |  |  |  |  |  |  | 5 (15.2) |
|  | need to justify own decisions regarding COVID-19 measures to others | **N** |  | **+** |  |  |  |  |  |  |  |  |  |  |  |  |  |  |  |  |  |  | # |  |  |  |  |  |  |  |  |  |  |  | 3 (9.1) |
|  | perspective regarding government work |  |  | **N** | **+** | + | + |  |  |  |  |  | + |  |  | + |  |  |  |  | + |  | + | + | + | # |  |  |  | + | + | + | + |  | 15 (45.5) |
|  | reasons for accepting the measures |  |  | **N** | **+** |  |  |  |  |  |  |  |  |  | + |  | + |  |  |  |  |  |  |  |  |  |  |  |  |  |  |  |  |  | 4 (12.1) |
|  | relief through the easing of measures | **#** |  | **#** | **#** |  |  | **#** | # | **#** | # | # |  | **#** | # | # | # |  |  | **N** | + | + | # | + |  |  | # | + | + | + | # |  | # | # | 24 (72.7) |
|  | trust in information provided |  |  |  |  |  |  |  |  |  |  |  |  |  |  |  |  |  | **N** | + |  |  | + | + | + | + | + |  |  | + |  | + |  |  | 9 (27.3) |
|  | weighing up the amount of news so as not to be misinformed or overwhelmed; bored or frightened |  |  |  |  |  |  |  |  |  |  |  |  |  | **N** |  |  |  | + |  | + |  |  | + |  |  | + |  |  | + | + | + | + | + | 10 (30.3) |
|  | worries about the economic situation |  | **N** | + | **+** |  |  |  | **+** |  |  |  |  | **+** |  |  |  |  | + |  |  |  | + |  |  |  | + |  |  | + | + |  | # |  | 11 (33.3) |
|  | **Number of lower-level concepts addressed in an initial interview for the first time** | **+14** | **+8** | **+6** | **+0** | **+2** | **+0** | **+0** | **+2** | **+2** | **+0** | **+1** | **+1** | **+1** | **+3** | **+0** | **+0** | **+0** | **+1** | **+1** | **+0** | **+0** | **+1** | **+1** | **+0** | **+0** | **+0** | **+0** | **+0** | **+0** | **+0** | **+0** | **+0** | **+0** |  |

*Note. N = lower-level concept addressed in an initial interview for the first time; + = lower-level concept which had already been addressed in an initial interview before; # = lower-level concept addressed in a follow-up interview*

**Supplemental table C.** Key characteristics of each participant

| **No** | **Age, years** | **Sex** | **ISCED level** | **Employment status** | **Living situation** | **Underlying disease** |
| --- | --- | --- | --- | --- | --- | --- |
| 1 | 82 | female | 4,5 | retired | alone | malignancies |
| 2 | 47 | female | 2,3 | unemployed | with others | nervous system |
| 3 | 65 | female | 4,5 | retired | alone | respiratory system |
| 4 | 62 | female | 4,5 | retired | with others | respiratory system |
| 5 | 64 | female | 4,5 | retired | alone | nervous system |
| 6 | 76 | male | 4,5 | retired | with others | endocrine, nutritional, and metabolic system |
| 7 | 67 | female | 4,5 | retired | alone | - |
| 8 | 52 | female | 4,5 | full-time | with others | musculoskeletal system and connective tissue; respiratory system |
| 9 | 73 | female | 2,3 | retired | care facility | cardiovascular system; musculoskeletal system and connective tissue |
| 10 | 79 | female | 2,3 | retired | care facility | malignancies; musculoskeletal system and connective tissue |
| 11 | 79 | female | 2,3 | retired | care facility | cardiovascular system; eye, ear and related structures; musculoskeletal system and connective tissue |
| 12 | 92 | female | 4,5 | retired | care facility | musculoskeletal system and connective tissue |
| 13 | 80 | female | 2,3 | retired | care facility | eye, ear and related structures; digestive system; nervous system; |
| 14 | 82 | female | 4,5 | retired | care facility | endocrine, nutritional, and metabolic system; musculoskeletal system and connective tissue; nervous system |
| 15 | 85 | female | 4,5 | retired | with others | musculoskeletal system and connective tissue; respiratory system |
| 16 | 78 | female | 6,7,8 | retired | with others | malignancies; musculoskeletal system and connective tissue |
| 17 | 79 | male | 6,7,8 | retired | with others | cardiovascular system; eye, ear and related structures; musculoskeletal system and connective tissue; nervous system |
| 18 | 80 | female | 4,5 | retired | alone | musculoskeletal system and connective tissue |
| 19 | 90 | male | 4,5 | retired | care facility | malignancies; musculoskeletal system and connective tissue |
| 20 | 76 | male | 6,7,8 | retired | with others | cardiovascular system; endocrine, nutritional, and metabolic system; eye, ear and related structures |
| 21 | 74 | female | 4,5 | retired | with others | cardiovascular system; respiratory system¸ urogenital system |
| 22 | 78 | female | 4,5 | retired | alone | - |
| 23 | 46 | female | 6,7,8 | part-time | with others | respiratory system |
| 24 | 74 | male | 4,5 | retired | with others | cardiovascular system |
| 25 | 72 | female | 4,5 | retired | with others | cardiovascular system |
| 26 | 66 | female | 4,5 | part-time | alone | musculoskeletal system and connective tissue |
| 27 | 85 | male | 4,5 | retired | care facility | cardiovascular system; respiratory system |
| 28 | 81 | female | 2,3 | retired | with others | cardiovascular system; eye, ear and related structures, musculoskeletal system and connective tissue¸ nervous system |
| 29 | 75 | male | 4,5 | retired | with others | cardiovascular system; endocrine, nutritional, and metabolic system |
| 30 | 68 | female | 4,5 | retired | with others | cardiovascular system; endocrine, nutritional, and metabolic system |
| 31 | 70 | female | 4,5 | retired | with others | musculoskeletal system and connective tissue |
| 32 | 84 | male | 4,5 | retired | with others | cardiovascular system; endocrine, nutritional, and metabolic system; eye, ear and related structures; malignancies; musculoskeletal system and connective tissue |
| 33 | 71 | female | 4,5 | retired | with others | cardiovascular system |

*Note. ISCED = International Standard Classification of Education*
